# Comparison between Wild and Hatchery Populations of Korean Pen Shell (*Atrina pectinata*) Using Microsatellite DNA Markers

**DOI:** 10.3390/ijms12096024

**Published:** 2011-09-16

**Authors:** Hye Suck An, Byeong Hak Kim, Jang Wook Lee, Chun Mae Dong, Shin Kwon Kim, Yi Cheong Kim

**Affiliations:** New Strategy Research Center, National Fisheries Research and Development Institute, Busan 619-705, Korea; E-Mails: bhkim@nfrdi.go.kr (B.H.K.); lee9952@nfrdi.go.kr (J.W.L.); ehdcnsao@nfrdi.go.kr (C.M.D.); ksk4116@nfrdi.go.kr (S.K.K.); yckim@nfrdi.go.kr (Y.C.K.)

**Keywords:** Korean pen shell, *Atrina pectinata*, microsatellite, genetic marker, genetic differentiation, polymorphism

## Abstract

Pen shell (*Atrina pectinata*) is a popular food source with a high commercial value in a number of Asian Pacific areas. The natural *A. pectinata* population has been declining continuously over the past several decades. Microsatellite DNA markers are a useful DNA-based tool for monitoring the genetic variation of pen shell populations. In this study, 20 polymorphic microsatellite (MS) DNA markers were identified from a partial genomic pen shell DNA library enriched in CA repeats, and used to compare allelic variation between wild and hatchery pen shell populations in Korea. A total of 438 alleles were detected at the 20 MS loci in the two populations. All loci were easily amplified and demonstrated allelic variability, with the number of alleles ranging from 5 to 35 in the wild population and from 5 to 22 in the farmed population. The average observed and expected heterozygosities were 0.69 and 0.82, respectively, in the hatchery samples and 0.69 and 0.83, respectively, in the wild samples. Statistical analysis of fixation index (*F*_ST_) and analysis of molecular variance (AMOVA) showed minor, but significant, genetic differences between the wild and hatchery populations (*F*_ST_ = 0.0106, CI_95%_ = 0.003–0.017). These microsatellite loci may be valuable for future aquaculture and population genetic studies for developing conservation and management plans. Further studies with additional pen shell samples are needed to conclusively determine the genetic diversity between the wild and hatchery populations.

## 1. Introduction

Marine shells of the Pinnidae family are a popular food source with a high commercial value in a number of Asian Pacific areas. The pen shell, *Atrina pectinata* (Linnaeus 1767), belongs to the Pinnidae family and is a large (shell length up to 30 cm) suspension-feeding bivalve common along the coasts of Korea, Japan, and China [1]. *A. pectinata* is an infaunal bivalve found in habitats ranging from muddy to sandy sediment and from tidal flats to shallow subtidal environments up to 20 m in depth [2]. In Korea, the fishery operates primarily in the western coastal areas. In the 1990s, total catches of ~8000 tons of pen shell were harvested annually. Since then, the commercial catch of this clam has decreased continuously for a number of years, reaching a historical minimum of ~2,000 tons in the year 2000, and it has remained at low levels ever since [3]. The reasons for the decline of this fishery are unknown, although habitat loss resulting from coastal area development and over-fishing may be contributing factors. The decline has prompted increased interest in both artificial breeding practices and information regarding the genetics of pen shell populations for sustainable fishing. Pen shell aquaculture is extensive in southern Korea, especially in Jangheung. However, hatchery production has raised questions about the genetic differences between wild and hatchery populations and concerns regarding the maintenance of genetic diversity among cultured stocks. The reduced genetic diversity observed in most hatchery stocks could result in a loss of genetic variation, thereby reducing the ability of the population to adapt to new environments [4,5]. Thus, understanding patterns of genetic variation is necessary for successful aquaculture management and the preservation of aquatic biodiversity in the sustainable development of marine fisheries [6].

With the rapid development of pen shell aquaculture and breeding projects, molecular markers for studying the genetic variation can help elucidate the genetic differences among wild populations, assess genetic variation within captive stocks, and determine the genetic impacts of aquaculture on wild populations, thereby promoting sustainable aquaculture. Because of their high degree of variability, microsatellite (MS) DNA markers or simple sequence repeats (SSRs) are molecular markers that are suitable tools for monitoring changes in the genetic variation of farmed stocks, assigning parentage, and evaluating the genetic diversity and structure of various marine species for the improvement of fisheries and resource conservation [7–10]. Despite the high commercial interest in pen shell in Korea, no study has focused on the genetic variability and population structure of this species. Currently, a limited number of MS DNA markers are available for pen shell [11,12]. However, the statistical power depends not only on the number of scored loci but also on other factors, such as the degree of polymorphism of each locus and the sample size, and the use of a limited number of loci might fail to compile the most effective marker-panels for the identification of individuals. Therefore, many of the current MS markers need to be developed and screened to identify loci that are the most informative for various other applications, including studies of genome mapping, parentage, kinships and stock structure. The present study is aimed at identifying new microsatellite loci and examining the similarities and differences between wild and hatchery pen shell populations in Korea.

## 2. Results and Discussion

### 2.1. Microsatellite Markers Isolation

In total, more than 400 white colonies were obtained from the transformation with the Korean pen shell (CA)*_n_*-enriched genomic DNA library, 150 of which were screened by PCR for the presence of a repeat-containing insert. Sequencing of the inserts from these 150 colonies revealed 90 loci containing MS arrays with a minimum of five repeats, corresponding to an enrichment efficiency of 22.5%. These were primarily 2-bp repeat motifs, some of which were combined with other 2-bp repeat motifs. Primers were designed and tested for 43 loci that exhibited adequately long (>20 bp) and unique sequence regions flanking the MS array. Forty-seven loci were discarded because the MS sequences were so close to the linker sequence that primer sequences could not be designed for amplification. After initial PCR assays, only 21 primer sets successfully yielded variable profiles. The remaining 22 primer sets gave either inconsistent or no PCR products, despite adjusting the dNTP concentrations and using an annealing temperature gradient. An initial evaluation of the polymorphic status of each locus was done by genotyping in 16 individuals randomly selected from the wild population. With the exception of KAp25, which had one allele, all loci were polymorphic and showed different degrees of variability. The primer sequences, repeat motifs, annealing temperatures, fluorescent labels, and GenBank accession numbers for the 21 newly identified MS loci are summarized in Table 1.

Generally, MS DNA loci isolation by screening of genomic libraries using repetitive probes is tedious but can result in the acquisition of numerous MS DNA loci. In case of magnetic bead-based enrichment, the types and ratios of biotin-labeled probes and the positive clone selection strategy can affect the success of cloning and the efficiency of enrichment. In this study, we created MS libraries enriched for CA repeat sequences by following the protocol of Hamilton *et al*. [13] with modifications that have been previously described [14,15]. Of the positive clones obtained, about 22.5% contained microsatellite repeats (90 of 400); this number is comparable with numbers obtained for *Pinctada maxima* (25%) [16], but lower than numbers obtained for *Haliotis midae* (47%) [17] and *Mercenaria mercenaria* (59.4%) [18]. Except for the efficiency of enrichment procedure, the differences in enrichment efficiency are probably a result of the use of different biotin-labeled oligonucleotide probes and the proper ratio. However, some studies have also suggested remarkable differences in microsatellite density among closely related species [19].

### 2.2. Genetic Variation within Populations

Samples of 63 wild and 50 hatchery-bred *A. pectinata* collected from around Jangheung, Korea, were screened for variation at the 20 new polymorphic MS loci. The 20 primer sets yielded variable profiles; reruns were conducted for 20% of the samples to ensure that the allele scoring was reproducible. No differences were observed, indicating that genotyping errors did not affect allele scoring. Samples that failed to amplify after the rerun were not included, which made it unlikely that poor DNA quality affected our results.

The MICRO-CHECKER analysis showed that some loci may have been influenced by one or more null alleles in both the wild and hatchery samples. Our data showed that the loci, KAp12, KAp21, KAp23, KAp32, KAp33, KAp40, and KAp43, in the farmed samples and the loci, KAp18, KAp21, KAp23, KAp26, KAp33, KAp40, and KAp43, in the wild population were affected. The KAp21, KAp23, KAp33, KAp40, and KAp43 loci appeared to be influenced in both the wild and hatchery samples, indicating that the use of these particular loci for population genetics analyses that assume HWE may be problematic. Thus, a global multi-locus *F*_ST_ value was estimated with and without these loci. With KAp12, KAp18, KAp26, and KAp32, however, our data indicated that these loci were affected by null alleles in only one sample; thus, they were included in further analyses.

All 20 MS loci were found to be highly polymorphic in both populations. A total of 438 different alleles were observed and the average number of alleles per locus was 21.9. The number of alleles varied from five at the KAp30 and KAp42 loci to 35 at the KAp21 locus (Table 2). Not all loci were equally variable. Especially, KAp12, KAp21 and KAp38 displayed greater allelic diversity, as well as higher levels of heterozygosity. The observed heterozygosity ranged from 0.111 at the KAp21 locus to 0.960 at KAp11, whereas the expected heterozygosity varied from 0.285 at KAp30 to 0.949 at KAp11 (Table 2). Due to the difference in sample size of the wild and hatchery populations, the parameter allelic richness (*A*_R_) was employed to compare different populations independent of sample size. Overall allelic richness varied from 4.59 to 32.11 (Table 2). A higher mean allelic richness was observed in the wild population (*A*_R_ = 17.41) than the hatchery population (*A*_R_ = 17.05) based on minimal sample size of 50. The wild population had a higher number of alleles and a higher allelic richness than the hatchery-bred population, but the difference was not significant (Wilcoxon signed-rank test, *P* > 0.05). The mean observed and expected heterozygosities were respectively 0.689 and 0.825 in the wild samples while the corresponding parameters were 0.684 and 0.822 in the hatchery samples, respectively. There were not significant differences of expected and observed heterozygosity between the wild and hatchery populations (Wilcoxon signed-rank test, *P* > 0.05).

In this study, a high level of genetic diversity (mean heterozygosity = 0.83; mean allelic number = 18.7) in the wild population was detected. A similarly high genetic diversity has been reported in many other marine bivalves such as oyster [20], clam [21], and scallop [22], indicating that it is a common characteristic of bivalves. Large population sizes and high nucleotide mutation rates are possible explanations for this high level of diversity [23].

Inbreeding coefficients (*F*_IS_) varied among markers from −0.144 (KAp30) to 0.883 (KAp21) in the hatchery samples and from −0.023 (KAp39) to 0.574 (KAp23) in the wild samples. The average *F*_IS_, including all markers, was 0.170 in the hatchery samples and 0.166 in the wild samples (Table 2).

**Figure 1:**
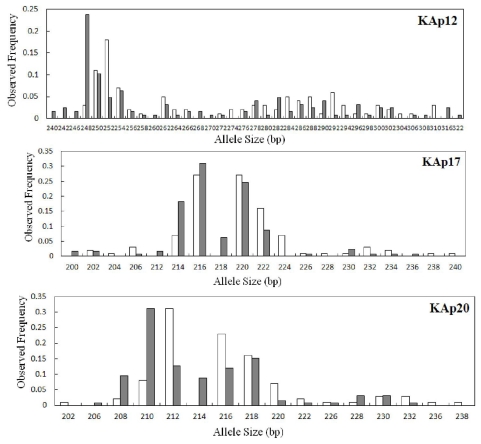

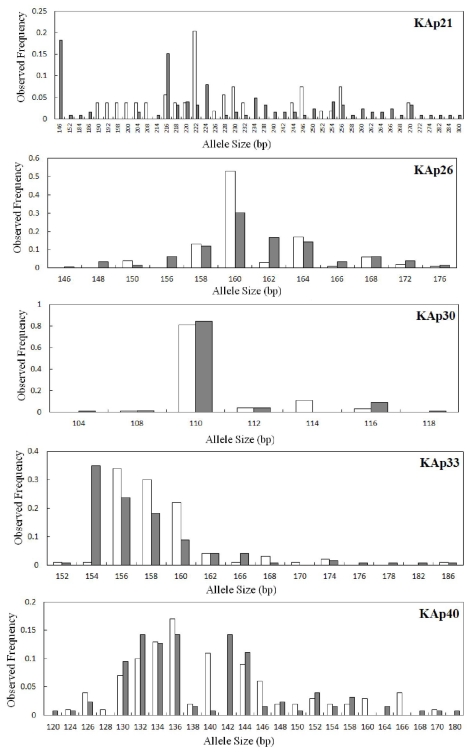
Allele frequency distributions at the eight microsatellite loci which showed significant heterogeneity from the wild (closed box) and hatchery (open box) populations of *Atrina pectinata* used in this study.

Table 3 shows the results of a heterogeneity test of allele frequency distributions in wild and hatchery samples. Two samples showed significant heterogeneity in allele frequencies for at least eight loci even after sequential Bonferroni correction for multiple tests (*P* < 0.003). The allele frequencies of the eight microsatellites in the wild and hatchery samples are shown in Figure 1. The maximum number of alleles was detected at locus KAp21 (*n* = 42), and the allele frequencies at this locus were clearly different between the wild and hatchery populations. Distinct differences in the allele frequencies between the wild and hatchery populations were also observed at the loci, KAp12, KAp17, KAp20, KAp26, KAp30, KAp33, and KAp40. More unique alleles were observed in the wild population (96) than in the hatchery population (64). Allele frequency distributions indicated the presence of 217 rare alleles (frequency <5%) out of a total of 341 alleles over all loci (63.64% rare alleles) in the hatchery sample, whereas 263 rare alleles out of a total of 374 alleles (70.32% rare alleles) were observed in the wild sample. Rare alleles were detected at most loci in both populations. Examination of pair-wise linkage disequilibrium revealed that all of the 20 microsatellite loci were in linkage equilibrium (*P* > 0.003).

In testing the departure from mutation-drift equilibrium based on heterozygosity excess or deficiency for both populations, bottleneck analysis was conducted using Bottleneck software under the two-phased model of microsatellites. No population displayed significant heterozygosity excess (*P* > 0.05) through the sign test, standardized differences test and Wilcoxon sign rank test, suggesting that both the wild and hatchery populations have not experienced a recent bottleneck.

Significant departures from HWE after Bonferroni correction (*P* < 0.003) were found at seven loci (KAp12, KAp21, KAp23, KAp32, KAp33, KAp40, and KAp43) in the hatchery samples and seven loci (KAp18, KAp21, KAp23, KAp26, KAp33, KAp40, and KAp43) in the wild samples, indicating that deviations from HWE detected at nine of the 20 microsatellite loci were due to heterozygote deficiency.

In the hatchery populations, homozygote excess is commonly caused by a limited number of founders or founder effects. In the wild populations, homozygote excess could be explained by a population effect such as the Wahlund effect or inbreeding or the effective population size and other potential causes such as nonamplifying alleles. However, in this study, the lack of significant differences between samples in their genetic diversity and the absence of significant heterozygosity excess, according to Bottleneck software, do not support different explanations for the homozygote excess observed at hatchery and wild samples. Thus, the homozygote excess observed in hatchery and wild populations is probably caused by null alleles, a locus-dependent effect found frequently at MS DNA loci. Significant heterozygote deficiency has previously been reported in marine invertebrate species [25–28], and it has been found that null alleles are the most likely cause of the heterozygote deficiency in HWE tests [29]. Indeed, our MICRO-CHECKER analysis suggested the presence of null alleles at loci with a significant heterozygote deficit. Furthermore, the widespread occurrence of null alleles has also been reported in other marine bivalves such as oyster [20] and scallop [30].

This study was limited by the number of populations screened. The genetic diversity parameters for each population as well as the HW disequilibrium at KAp18 and KAp26 observed in the wild samples may be explained by data from additional populations, which may allow for a more precise genetic characterization of the MS loci used. Therefore, our results should be interpreted with caution. Further study is required to assess the genetic resources of wild populations and the influence of aquaculture on the genetic structure of this important fishery species.

### 2.3. Genetic Diversity between the Wild and Hatchery Populations

Single-locus *F*_ST_ estimates and global multi-locus *F*_ST_ values were significantly different between the hatchery and wild populations. The *F*_ST_ value, including all loci, was estimated to be 0.0162 (*P* < 0.01). When the KAp21, KAp23, KAp33, KAp40, and KAp43 loci were excluded, the global multi-locus *F*_ST_ was estimated to be 0.0106 (*P* < 0.01).

The analysis of molecular variance (AMOVA) of the 15 microsatellites yielded results similar to those from the FSTAT analysis; variation within individuals, among individuals within populations, and among populations was 93.32% (*P* = 0.000), 5.67% (*P* = 0.000), and 1.06% (*P* = 0.006), respectively (Table 4). *F*_IS_, *F*_ST_, and *F*_IT_ were 0.057, 0.011, and 0.066, respectively. AMOVA showed that the variation between the wild and hatchery populations explained only 1.06% of the total variance, whereas the variation among individuals within populations and within individuals explained 5.67% and 93.32% of the total variation, respectively.

Statistical analysis of fixation index (*F*_ST_) and analysis of molecular variance (AMOVA) showed minor, but significant, genetic differentiation between the wild and hatchery populations. This is confirmed by the allele frequency distributions and the numbers of unique allele and rare allele in each MS locus between the wild and hatchery populations. In pen shell populations in Jangheung, Korea, the progeny produced for stock abundance had a different genetic composition although no significant reductions were found in the diversity compared with the wild population (*P* > 0.05; Table 2). In fact, the loss of alleles is more important than a change in allele frequencies because the latter can be changed again by random drift, but there is no way to recover a lost allele. This difference in allele number is probably a result of reduced genetic variation due to hatchery selection and inbreeding. Continued hatchery reproduction might lead to considerably decreased genetic variability. The limited genetic differentiation between the wild and hatchery populations may suggest a relatively short domestication history of hatchery pen shell. Several studies have reported a marked reduction in genetic variability at MS loci in hatchery populations and a reduced fitness in hatchery-bred individuals when exposed to natural environments [31–33]. This reduced variability is likely due to the limited numbers of effective breeders used for reproduction.

## 3. Experimental Section

### 3.1. Sample Collection and DNA Extraction

Mantle musculature was collected from an individual pen shell from Jangheung, Korea, which was used for genomic DNA isolation of high-molecular-weight and microsatellite enriched partial genomic library construction. Samples of 63 wild and 50 hatchery-bred pen shells were collected from Jangheung, Korea, in July 2008. Pen shells were sampled from a wild population in a tidal area. The hatchery population samples were randomly selected from one broodstock population, which is derived from the founders originally collecting from the wild population. All the samples were placed in absolute ethanol and kept frozen at −20 °C until DNA extraction. The TNES-urea buffer method [34] was used to isolate high-molecular-weight DNA for microsatellite isolation. For genotyping, total DNA from mantle-clips of each sample was extracted using a MagExtractor-Genomic DNA Purification Kit (TOYOBO, Osaka, Japan) for an automated DNA extraction system, MagExtractor MFX–2100 (TOYOBO). Extracted genomic DNA (20 μg) was stored at −20 °C until further use for PCR.

### 3.2. Genomic Library Construction and Microsatellite Sequencing

A partial genomic library enriched for CA repeats was constructed using a slightly modified enrichment procedure with pre-hybridization polymerase chain reaction (PCR) amplification, as described previously [12]. The extracted DNA was digested with the restriction enzymes *Alu*I, *Rsa*I, *Nhe*I, and *Hha*I (New England Biolabs, USA). DNA fragments in the range of 300–800 bp were isolated and purified using the QIAquick Gel Extraction Kit (Qiagen, Germany). The selected fragments were ligated to an adaptor (SNX/SNX rev linker sequences), and the linker-ligated DNA was amplified using SNX as a linker-specific primer for PCR. For enrichment, the DNA was denatured and biotin-labeled repeat sequences ((CA)12GCTTGA) [35] were hybridized to the PCR products. The hybridized complex was separated with streptavidin-coated magnetic spheres (Promega, USA). After washing, the bound, enriched DNA was eluted from the magnetic spheres and re-amplified with an adaptor sequence primer. PCR products were purified using a QIAquick PCR Purification Kit (Qiagen, Germany).

The purified PCR products were digested with NheI, cloned using an XbaI-digested pUC18 vector (Pharmacia, USA), and transformed into *Escherichia coli* DH5 competent cells. White colonies were screened for the presence of a repeat insert by PCR using the universal M13 primer and non-biotin-labeled dinucleotide primers. PCR products were examined on 2% agarose gels, and inserts producing two or more bands were considered to contain a microsatellite locus. Positive clones were cultured and purified. Plasmids from insert-containing colonies were recovered using the QIAprep Spin Miniprep Kit (Qiagen, Germany) and sequenced using the BigDye Terminator Cycle Sequencing Ready Reaction Kit (ver. 3.1; Applied Biosystems, USA) and an automated sequencer (ABI Prism 310 Genetic Analyzer, Applied Biosystems).

### 3.3. Primer Design and Allele Scoring

Primers were designed based on sequences flanking the MS motifs using the OLIGO software package (ver. 5.0; National Biosciences, USA). Newly designed PCR primer pairs were tested to optimize the annealing temperatures; a gradient PCR with a 50–60 °C range was performed on a sample set from eight pen shells captured from Jangheung, Korea. The PCR amplification was performed using a PTC 200 DNA Engine (MJ Research, USA) in a 10-μL reaction containing 0.25 U of *Ex Taq* DNA polymerase (TaKaRa Biomedical, Japan), 1× PCR buffer, 0.2 mM dNTP mix, 100 ng of template DNA, and 10 pmol of each primer, where the forward primer from each pair was 5′-end-labeled with 6-FAM, NED, and HEX dyes (Applied Biosystems). The PCR reaction ran for 11 min at 95 °C, followed by 35 cycles of 1 min at 94 °C, 1 min at the annealing temperature (Table 1), and 1 min at 72 °C, with a 5-min final extension at 72 °C. Microsatellite polymorphisms were screened using an ABI PRISM 3100 Automated DNA Sequencer (Applied Biosystems) and alleles were designated by PCR product size relative to a molecular size marker (GENESCAN 400 HD [ROX], Applied Biosystems). Fluorescent DNA fragments were analyzed using the GENESCAN (ver. 3.7) and GENOTYPER (ver. 3.7) software packages (Applied Biosystems, USA).

### 3.4. Sample Comparisons

Samples were screened for variation at the newly developed MS loci. MICRO-CHECKER 2.2.3 [36] was used to detect genotyping errors due to null alleles, stuttering, or allele dropout using 1000 randomizations. For genetic diversity parameters, the number of alleles per locus (N_A_), size of alleles in base pairs (S), frequency of the most common allele (F), and number of unique alleles (U) were determined for each local sample at each locus using the program GENEPOP (ver. 4.0; http://kimura.univ-montp2.fr/~rousset/Genepop.htm). This was also used to identify deviation from Hardy-Weinberg equilibrium (HWE; exact tests, 1000 iterations) and the observed and expected heterozygosities, indicating an excess or deficiency of heterozygotes. FSTAT (ver. 2.9.3.2; http://www.unil.ch/izea/softwares/fstat.html) was used to calculate the inbreeding coefficient (*F*_IS_) [37] per locus and sample and allelic richness (*A*_R_) [38], suitable for comparing the mean number of alleles among populations regardless of sample size. For analysis of molecular variance (AMOVA) [39], components of variance within and between populations based on the infinite allele model (IAM) were estimated using the ARLEQUIN program (ver. 3.0) [40]. The significance of AMOVA components was tested using 1,000 permutations. ARLEQUIN was also used to assess linkage disequilibrium for all pairs of loci, whose empirical distribution is obtained by a permutation procedure [41] and to calculate single-locus and global multilocus values (*F*_ST_; 1,000 permutations) [37]. Significance levels were adjusted for multiple tests by using sequential Bonferroni correction [24]. As hatchery populations are often subjected to founder effects and bottleneck that result in lower genetic diversity, Bottleneck software (ver. 1.2.02) [42] was employed to test the bottleneck hypothesis under a two-phased model of mutation (TPM). This method can be used for testing the departure from mutation-drift equilibrium based on heterozygosity excess or deficiency.

## 4. Conclusions

In conclusion, genetic studies on pen shell with microsatellite DNA markers are very rare with only two existing reports on the development of new microsatellites [11,12]. No detailed information is available to date on the genetic diversity of wild and cultured stocks of pen shell. In this study, we report the identification of new microsatellite loci in pen shell. Twenty highly polymorphic and quality microsatellite loci were characterized and used to study genetic differences between the wild and hatchery populations. A total of 438 alleles were detected from the two populations, with 374 alleles detected in the wild population and 341 detected in the hatchery population. Minor but significant genetic differences were detected between the wild and hatchery populations based on an analysis of 15 microsatellite loci during which no null allele was detected. Continued monitoring of genetic variance between wild and hatchery pen shell stocks through DNA markers is essential for the successful implementation of an artificial pen shell breeding project and the conservation of natural Korean pen shell resources.

## Figures and Tables

**Table 1:** Characteristics of the 21 microsatellite loci isolated from *Atrina pectinata*.

Locus	Repeat Motif	Primer Sequence (5′→3′)	*T*_a_ (°C)	*Genebank Accession No.*
KAp2	(GT)_12_	F: CAATGTTGATGATGGATGTTA **ned** R: GCTTCATGTGGGTTTGG	*60*	EU026353
KAp6	(GT)_12_	F: CAGCTACCAAAACCATAAATC **hex** R: CCAGTCAGCTTGAGTTACAGA	*60*	EU026354
KAp9	(GT)_9_	F: AATGGTACAGTTGTACAGCAC **ned** R: CAAGCATATTTGTCATTTGAT	*55*	EU026355
KAp11	(CA)_12_	F: GCTGTTGGAAATACGGACTAC **6-fam** R: CCACCAAAATTCGGTAAAA	*55*	EU026356
KAp12	(GT)_13_	F: CGATCCTATCCAAGGGTTAT **6-fam** R: CTCTATCCGTTCTCCATTTCT	*55*	EU026357
KAp17	(CA)_11_	F: GCAAGGCAAAATGTATTACC **6-fam** R: CGAGTACTTGCCGTAGTGAC	*55*	EU026358
KAp18	(CA)_12_	F: TTGGAAATACGGACTACTCA **hex** R: CCTTGACGTGACCACTTA	*57*	EU026359
KAp19	(GT)_15_	F: CAAAATCCAGGAGTATCTCA **hex** R: TTCCCTAGAGTGCATAAACT	*55*	EU026360
KAp20	(GT)_11_	F: ACCGTAGTGACACTGAAGGA **hex** R: GAGGCAAGGCAAAATGTAT	*55*	EU026361
KAp21	(GT)_15_	F: GGAATCATTCTCGCAATA **6-fam** R: GAAGCACGTATCATCACTAA	*55*	EU026362
KAp23	(CA)_11_	F: ATCAAGTCATTGCCACAC n**ed** R: AGAAGCACTTGCCGTAG	*57*	EU026363
KAp24	(GT)_13_	F: CCGTGCTGTGGTAATGTA **hex** R: TTGGCATAAATAGAAAGGTT	*52*	EU026364
KAp25	(CA)_22_	F: CGCGTTCGACTCTTGA n**ed** R: CAAAATTTGGCCTATGCT	*52*	EU026365
KAp26	(GT)_10_	F: GGCGTGTCTATACTTGAACT **6-fam** R: TGTCACTTGCGTCACTTTA	*55*	EU026366
KAp30	(GT)_11_	F: TTGAACAAAGACTTGTCA n**ed** R: TCACGTTGAGACTTCATA	*52*	EU026367
KAp32	(CA)_13_	F: TCATCATGTGGCTGTATA n**ed** R: CTGCAGTTGCTTGAAG	*55*	EU026368
KAp33	(GT)_14_AA(GT)_4_	F: CCACCTGACTGTCTCTGA **hex** R: ACAGAATCTCGCCTAAAG	*52*	EU026369
KAp38	(GT)CT(GT)_13_	F: CGCCTACATTTAGTCAGT n**ed** R: CTTGGCTTGTACCATATC	*50*	EU026370
KAp39	(GT)_21_T(GT)	F: CCGACGTATTATTAGTGC **6-fam** R: GCGTAACCCATGTATTAA	*50*	EU026371
KAp40	(GT)_10_TT(GT)_4_	F: CCATTCACCAAGAGGTTG n**ed** R: CCGTGCGTTGTCGTAC	*50*	EU026372
KAp42	(GA)_3_(GT)_8_	F: GATAGGCTTCCGTTGTTCTA **hex** R: AGCCTATGTTCCACGAGAC	*55*	EU026373
KAp43	(TG)_8_	F: AGCTGTTTCACTCTCATTT **6-fam** R: AGAATTTTAACCACAACCTT	*52*	EU026374

*T*_a_ is the optimal annealing temperature.

**Table 2:** Summary of the statistics for 20 microsatellite loci in the two *Atrina pectinata* populations.

Microsatellite loci	*F*st	Population (No)																	
		Jangheung Wild (63)									Jangheung Hatchery (50)								

		*N*_A_	*A*_R_	*S*	*F*	*U*	*He*	*Ho*	*F*is	*P*	*N*_A_	*A*_R_	*S*	*F*	*U*	*He*	*Ho*	*F*is	*P*
KAp2	−0.0003	18	16.41	132–172	0.175	3	0.908	0.889	0.021	0.626	19	19.00	130–180	0.139	4	0.921	0.889	0.036	0.688
KAp6	0.0044	13	12.45	150–186	0.286	2	0.818	0.730	0.108	0.496	15	15.00	146–178	0.330	4	0.823	0.820	0.003	0.658
KAp9	−0.0045	17	15.5	100–150	0.278	7	0.849	0.794	0.066	0.702	14	14.00	100–138	0.330	4	0.823	0.820	0.004	0.067
KAp11	−0.0015	21	20.32	218–262	0.119	2	0.942	0.937	0.005	0.012	23	23.00	216–266	0.100	4	0.949	0.960	−0.011	0.500
KAp12	0.0294	34	31.19	240–322	0.238	9	0.920	0.873	0.051	0.564	28	28.00	248–310	0.180	3	0.938	0.840	0.106	0.000
KAp17	0.0102	14	12.84	200–236	0.310	4	0.803	0.714	0.112	0.214	15	15.00	202–240	0.270	5	0.824	0.880	−0.069	0.289
KAp18	0.0038	22	21.28	138–182	0.111	1	0.945	0.937	0.009	0.000	23	23.00	140–184	0.120	2	0.947	0.940	0.007	0.427
KAp19	0.0037	29	27.33	208–266	0.079	5	0.955	0.937	0.020	0.398	23	23.00	208–264	0.130	0	0.935	0.920	0.016	0.011
KAp20	0.0576	13	12.13	206–232	0.310	2	0.839	0.746	0.111	0.173	14	14.00	202–238	0.310	3	0.819	0.680	0.171	0.175
KAp21	0.0422	35	32.11	146–300	0.183	22	0.930	0.476	0.490	0.000	20	20.00	190–270	0.204	7	0.937	0.111	0.883	0.000
KAp23	0.0131	12	11.29	116–160	0.254	3	0.817	0.349	0.574	0.000	11	11.00	116–140	0.380	2	0.778	0.560	0.283	0.001
KAp26	0.0404	12	11.71	146–176	0.302	3	0.841	0.524	0.379	0.000	9	9.00	150–176	0.530	0	0.673	0.460	0.319	0.010
KAp30	0.0177	6	5.55	104–118	0.841	2	0.285	0.222	0.222	0.083	5	5.00	108–116	0.810	1	0.333	0.380	−0.144	1.000
KAp32	0.0049	23	21.43	126–208	0.206	5	0.917	0.825	0.101	0.625	26	26.00	126–196	0.190	8	0.924	0.740	0.201	0.000
KAp33	0.0861	13	11.72	152–186	0.349	3	0.783	0.571	0.272	0.000	11	11.00	152–186	0.340	1	0.750	0.440	0.416	0.000
KAp38	0.0077	32	29.28	80–146	0.246	10	0.915	0.873	0.047	0.373	24	24.00	78–144	0.200	2	0.906	0.840	0.074	0.072
KAp39	0.0041	19	17.24	186–248	0.444	5	0.760	0.778	−0.023	0.762	20	20.00	184–244	0.350	6	0.811	0.700	0.138	0.024
KAp40	0.0130	21	19.37	120–180	0.143	5	0.903	0.730	0.193	0.000	19	19.00	124–170	0.170	3	0.917	0.700	0.239	0.000
KAp42	−0.0040	5	4.59	184–200	0.659	1	0.497	0.397	0.203	0.049	6	6.00	172–202	0.610	2	0.534	0.500	0.065	0.594
KAp43	0.0027	15	14.46	224–274	0.254	2	0.875	0.476	0.458	0.000	16	16.00	226–268	0.210	3	0.900	0.500	0.447	0.000

Mean	0.0162	18.7	17.41		0.289	4.8	0.825	0.689			17.05	17.05		0.295	3.2	0.822	0.684		

Single-locus *F*_ST_, number of samples (No), number of alleles per locus (*N*_A_), allelic richness (*A*_R_), size of alleles in bp (*S*), number of unique alleles (*U*), expected heterozygosity (*H*_e_), observed heterozygosity (*H*o), inbreeding coefficient (*F*_IS_), and probability of a significant deviation from Hardy-Weinberg equilibrium after the Bonferroni correction (*P*, initial α = 0.05/20 = 0.003) are given for each population and locus.

**Table 3:** Comparison of allele frequencies between the wild and hatchery populations at 20 microsatellite loci of *Atrina pectinata*.

Locus	*P*-value	Locus	*P*-value
KAp2	0.383	KAp23	0.012
KAp6	0.083	KAp26	0.000*
KAp9	0.695	KAp30	0.001*
KAp11	0.575	KAp32	0.040
KAp12	0.001*	KAp33	0.000*
KAp17	0.000*	KAp38	0.292
KAp18	0.191	KAp39	0.486
KAp19	0.259	KAp40	0.000*
KAp20	0.000*	KAp41	0.797
KAp21	0.000*	KAp42	0.602

Probability values of homogeneity of allelic frequency distributions (*P*) estimated by a test analogous to the Fisher exact test in the Markov-chain method are shown; wide significance levels were applied using the sequential Bonferroni technique (*k* = 20) [24].

* Significant at *P* < 0.003.

**Table 4:** Analysis of molecular variance (AMOVA) of 15 microsatellite loci in the wild and hatchery populations of *Atrina pectinata*.

Source of Variation	Sum of Squares	Variance Components	Percentage Variation (%)	*P*-Value
Among Populations	11.881	0.524	1.059	0.006
Among individuals with population	663	0	5.668	0.000
Within individuals	602.000	5.383	93.323	0.000
Total	1277.273	5.761		
